# Redefining protein moonlighting

**DOI:** 10.18632/oncotarget.4793

**Published:** 2015-07-10

**Authors:** Charles E. Chapple, Christine Brun

**Affiliations:** Inserm, UMR1090 TAGC, Aix-Marseille Université, UMR_S1090 TAGC, CNRS, Marseille, France

We recently presented the first computational method for the large-scale identification of what we have termed “extreme multifunctional proteins” (EMF) [1]. These are proteins whose multiple functions are very dissimilar to one another. While obviously related to moonlighting proteins, we chose to coin a new term because we felt that the current definition of moonlighting is too constrictive.

The first use of “protein moonlighting” was by Constance Jeffery [2] in 1999 who defined such proteins as having multiple functions while excluding proteins that are the “result of gene fusions, homologous but non-identical, splice variants, proteins whose post-translational modifications can vary and proteins that have a single function but can operate in different locations or utilize different substrates”. A more recent review [3] defined them as “special multifunctional proteins, because they perform multiple autonomous, often unrelated, functions without partitioning these functions into different protein domains”.

We feel that a new, less restrictive definition is needed. What makes moonlighting proteins interesting is the fact that they perform multiple unrelated functions. The evolutionary history of the protein, whether it is the result of a gene fusion event or not, does not change the fact that in its present form the protein performs multiple unrelated functions. We believe that what makes these proteins worth studying is simply that they are involved in dissimilar processes, and that is what their definition should emphasize.

Current definitions require that a moonlighting protein's multiple functions not be partitioned into separate domains. This leads to two issues. First, domain identification is imperfect. It is often the case that we simply can't find the domain in question; either because it is below our detection thresholds or because it is novel and not present in the databases or literature. Second, proteins whose multiple functions are performed by separate domains are no less intriguing than those whose functions are performed by the same one. What makes these proteins so fascinating is that they combine such dissimilar functions, irrespective of their domain organization.

By sidestepping the issue and defining extreme multifunctionality, we were able to perform a large-scale search for such proteins in the human protein interaction network. We developed two measures of functional dissimilarity [4], one based on the frequency with which the two functions are performed by the same protein, the other based on the frequency of interactions between proteins performing each function. We combined these to define “dissimilar” functions. We then divided the interaction network into overlapping clusters of proteins, annotated each cluster in terms of the functions its constituent proteins perform (thereby identifying functional “modules”) and searched for proteins belonging to at least two clusters annotated to dissimilar functions. In this way, we identified 430 human extreme multifunctional proteins.

These proteins, our EMF candidates, shared characteristics that set them apart from the network, other multifunctional proteins (those that belonged to multiple clusters) and hubs. A typical EMF is likely to have a high number of interactors, to belong to more network modules and to be more central to the network. It is more likely to be involved in multiple diseases and to be expressed more ubiquitously, suggesting that it can perform different functions in different tissues. It will also have more domains, be more conserved than a classical multifunctional protein, and contain more short linear motifs (ELMs).

Two of these shared features are particularly interesting. First, the candidates were no richer in disordered regions than the network average. This is notable because it had previously been theorized [5] that disordered regions might help moonlighting proteins adopt different conformations, offering a possible explanation for their functional versatility. This is indeed true of network hubs which have been shown to be more disordered, on average, than non-hubs. Since the majority of our EMF candidates are also network hubs, that they have no more disorder than average suggests that, even among hubs, EMFs are different. A difference further emphasized by the fact that the disordered regions of EMFs are enriched in ELMs, short conserved sequences located in disordered regions, that might play regulatory roles by affecting low affinity protein binding [6]. These motifs therefore suggest possible molecular mechanisms that could explain moonlighting. If, as suggested by our findings, the moonlighting propensity of a protein is partly driven by ELMs, the relevance of the domain restriction in the original definition is further decreased.

In conclusion, we feel that it is time to extend the definition of moonlighting to simply “proteins whose multiple functions are unrelated”. Not only are such proteins intrinsically intriguing, they can also play an important role in many different and comorbid diseases as our results have shown [7]. Restricting the field of moonlighting proteins based on their evolutionary history and domain organization will, perforce, blind us to many important cases of extreme multifunctionality.
